# The sentinel behaviour of Arabian babbler floaters

**DOI:** 10.1098/rsos.160738

**Published:** 2017-02-08

**Authors:** Roni Ostreiher, Aviad Heifetz

**Affiliations:** 1 Department of Life Science, The Open University of Israel, The Dorothy de Rothschild Campus, 1 University Road, POB 808, Raanana 4353107, Israel; 2 The Open University of Israel, The Dorothy de Rothschild Campus, 1 University Road, POB 808, Raanana 4353107, Israel

**Keywords:** Arabian babbler, cooperative breeding, floater, sentinel, alarm calls, social hierarchy

## Abstract

The sentinel behaviour of 38 Arabian babbler adult floaters, who lived alone within a territory belonging to a foreign group, was studied and compared with their own sentinel behaviour in the past, when they were group members. All floaters acted as sentinels and uttered ‘alarm calls’. This suggests that sentinel activity is due at least, in part, to selfish motives. Floaters sentinelled less than they did as group members, with the decrease in sentinel activity sharper for ex-dominants than for ex-subordinates. One possible explanation for these differences is that sentinel activity is aimed not only at detecting predators, but also at detecting foreign conspecifics. Within a group, the latter incentive is stronger for breeding dominants than for subordinates, whereas all floaters alike may be trying to detect the owners of the territory in which they were roaming but also to avoid being detected by them. Other possible explanations are that floaters have less time and energy for sentinel activity because they are weaker or because foraging is more difficult in a foreign territory. This may be especially so for dominants who used to enjoy privileged access to food in their group. No significant difference was found in the rate of sentinels' ‘alarm calls’ between floaters and group members, suggesting that their main purpose is predator–prey communication, of which warning groupmates may be a side benefit.

## 1. Introduction

In some group-living animals, from time to time, one of the group members climbs up to a high position relative to the surroundings, perches steadily for a while and, to the human observer's eye, looks around. If a predator is approaching, then the observer utters special calls. Hearing them, other group members stop foraging immediately and escape into dense vegetation or climb to nearby treetops and also start calling. Many studies have agreed that this is a common system of anti-predator vigilance, occurring within stable groups of birds and mammals [1–14]. In the literature, the observer who perches higher than the other group members and looks around is called a ‘sentinel’, and its special calls when a predator approaches are termed ‘alarm calls’. Animal sentinel behaviour has been widely studied in many species of mammals, birds and even fish (reviewed by [15]). As can be imagined, many of them are cooperative breeders, such as jungle babblers, *Turdoides striatus* [1], pied babblers, *Turdoides bicolor*, [16,17], Arabian babblers, *Turdoides squamiceps* [5], Florida scrub jays, *Aphelocoma coerulescens* [4], white-browed sparrow weavers, *Plocepasser mahali* [18,19], dwarf mongooses, *Helogala parvula* [2,14] and meerkats, *Suricata suricatta* [20].

Usually, but not always, only one group member at a time acts as a sentinel. The sentinel activity covers just part of the group's foraging time and, for the Arabian babbler, between 40% and 70% of the group's foraging time is covered by a sentinel [9]. In the pied babbler, the time is about 30% [16]. White-browed sparrow weaver groups have a sentinel about 21% of the time [19]. In the Florida scrub jay, groups were guarded by at least one sentinel about 33% in late summer, 50% in spring and about 75% in winter [4]. In the meerkats, there is a sentinel 55.6% of the group's foraging time [20]. These examples show that the sentinel system's coverage is not comprehensive and that group members are not always defended by a sentinel when they are foraging.

The Arabian babbler, *T. squamiceps*, is a cooperatively breeding songbird [5,21], resident along the dry riverbeds of southeastern Israel. Arabian babblers live year round in territorial groups of 2–20 individuals, of both sexes and all ages. All group members cooperate in defending the territory against neighbouring groups and against intruding babblers who occasionally try to penetrate into the area or into the group. A linear hierarchy exists among individuals of each sex that typically corresponds to the original hatching order of the individuals [5,21]. Within the group, a higher rank implies both a higher priority for access to food as well as for breeding opportunities [5,21].

*Floaters* are individuals who live alone, inconspicuously, within a territory of a group without being members of that group (following [22,23]). They maintain all life activities, including foraging and roosting by themselves. Individuals become floaters in breeding populations when suitable habitats become saturated with dominant territory owners [22,24,25].

At our research site (see Methods), the area is saturated [5]: there is no free space which is suitable for babbler living and is not already occupied by babbler groups. Leaving one's territory means automatically and immediately entering the territory of a neighbouring group. Babbler groups are usually intolerant of the presence of foreign babblers in their territory. When ‘foreigners’ are discovered by the owners of the territory, they are chased away, and they have to move between territories. All floaters were group members in the past and became floaters either by being evicted from their group by another babbler of the same sex or by leaving the group without joining another one.

In the latter case, sometimes they can return to their original group and sometimes they cannot. They are not prospectors (individuals who visit other territories to display or to fight, but return to their own group between interactions until they attain a position in a new group, see [26,27]), because the duration of living out of their natal territory is relatively long (many days or even months), they maintain all life activities, including roosting, alone and when they are discovered by the owners of the territory, they are chased away and escape, but not necessarily back to their natal territory.

Over 28 years of study (1988–2015), we have encountered 131 Arabian babbler floaters. All of them were adults (older than 2 years) and 74 of them (56.5%) were females (unpublished data from 1988--2015). Some of them were observed only once or twice and disappeared from the research area, but some of them succeeded to survive as floaters for more than a year. Twenty of them (15.3%) succeeded in joining a group, seven (5.3%) established a new group, six (4.6%) returned to their previous group and the rest of them—98 babblers (74.8%)—disappeared from the research area and we do not know what their fate was (unpublished data from 1988--2015). We believe that in a population of about 160 individuals living in 25 groups, between two and six individuals (about 1–4%) live as floaters and move between the territories, but it is possible that we have underestimated the phenomenon.

Within groups, the sentinel behaviour of the Arabian babbler and its vocalization have already been studied in our research site by Zahavi [5], Zahavi & Zahavi [28], Naguib *et al*. [29], Wright *et al*. [8,9,30], Regosin [31], Sommer [32], Sommer *et al*. [33] and Dattner *et al*. [13]. However, the sentinel behaviour of *floaters* has not yet been studied in the Arabian babbler.

Unlike floaters' sentinel activity, which, to the best of our knowledge, has not yet been studied in other species either, floaters' vigilance behaviour was already studied in the pied babbler [27]. Vigilance is defined as scanning the surrounding area with head up rather than foraging with head lowered [34]. In particular, vigilance behaviour is frequently carried out on the ground, whereas sentinel activity requires assuming a high position. Moreover, vigilance behaviour is usually carried out for a few seconds [27], whereas sentinel activity is typically carried out for several minutes (see Results). Another difference is that sentinel activity is usually carried out by one individual at a time, while vigilance behaviour is frequently carried out simultaneously by more than one individual.

Bednekoff [15] defined sentinel behaviour as coordination of vigilance among group members. In the case of floaters, the coordination aspect is, of course, absent. In this study, we nevertheless maintain the term ‘sentinel’ for the case of the floaters as well, because for each such individual, we compare its sentinel behaviour when it was a group member (and coordination of vigilance was relevant) with its same physical behaviour (perching on a high branch, looking around and uttering ‘alarm calls’ in reaction to approaching predators) when that individual became a floater. Thus, for the particular case of floaters—who were all group members in the past—this usage of the term ‘sentinel’ need not cause confusion, and is more transparent than assigning different terms to the same physical behaviour in the social and solitary contexts.

The aim of this study is to compare the sentinel behaviour of Arabian babbler floaters with their own sentinel behaviour when they were group members in order to better understand this complex phenomenon. At the beginning of the study, we were surprised to discover that floaters carry out sentinel behaviour. We tested three alternative hypotheses (i) no difference would exist between the sentinel behaviour of floaters versus their own sentinel behaviour as group members, because there is no reason to assume that the dangers are different (the fact that they became floaters does not change the number of predators around); (ii) floaters would sentinel more than group members because they face a higher level of danger (nobody warns them when a predator is approaching) and (iii) floaters would sentinel less than group members because standing steady on the treetop in foreign territory reveals them to the owners who would likely attack them or, because they need more time to forage in a foreign territory, they have less time left for sentinel activity.

## 2. Methods

The study was carried out at the Shezaf Nature Reserve in the Arava Valley, in the southeastern part of Israel. The study area, the research population, the Arabian babbler's social system and our fieldwork methods have been described by Zahavi [21] and by Ostreiher [35,36].

The study area contained about 160–260 individuals who lived in 25–32 groups. Each group was observed at least twice a week and, in the breeding seasons, almost daily. The nestlings were ringed when they were 10 days old, 4 days before fledging. Each of them was ringed with four coloured rings in a unique combination, enabling us to identify them individually throughout their life. The babblers were accustomed to human presence, thus the researchers were able to stay in their vicinity as much as needed.

Over 22 years (1990–2011), we followed 38 adult Arabian babbler floaters who lived alone, outside of their original territory. Fifteen of them were males and 23 were females. All the floaters wore coloured rings which enabled identifying them individually, their exact age was known (±1 day) and they were accustomed to close human presence. We had many details about their behaviour as group members before they became floaters. Out of 38 floaters, before leaving their groups, 11 were the dominant babblers in their groups (number 1 in the social hierarchy, seven males and four females), 14 were number 2 (five males and nine females), 11 were number 3 (three males and eight females) and 2 were number 4 (both females). We defined their rank in the social hierarchy in the group according to aggressive/submissive interactions with their groupmates [5].

Out of these 38 floaters, 16 were observed being chased out of their groups (eight males and eight females). For 22 babblers (seven males and 15 females), we did not observe how they left the group.

We refer to these babblers as floaters and not as prospectors because they stayed alone in foreign territory and did not return to their natal groups for at least five consecutive days, and usually much longer, not even for roosting.

The 38 floaters hatched and grew up in 25 different groups. Thirteen floaters who left or were expelled from groups from which another floater had already moved away did so with a gap of at least 6 years and a completely different social composition of the group.

For many years, we used to observe a group for three consecutive days. Each day we carried out two 3 h observations. We started with the first light in the morning, and the second was carried out in the afternoon, ending at darkness. The behaviour of all group members was documented, including, in particular, the number of sentinel events of each individual, the duration of sentinel events, the location of the sentinel and its behaviour as well as the behaviour of other group members. This method of data collection was also kept for floaters. When a floater was found in the research area, we followed it over three consecutive days and documented its behaviour. Every day, we maintained two 3 h observations: one in the morning, starting with the first light and the second in the afternoon, ending at darkness.

An individual was defined in this study as carrying out sentinel activity when it climbed to a high position above the ground (usually on a treetop), stood steady and looked around without foraging until it left its position. The starting and ending time of each sentinel event was measured with a stopwatch with 1 s accuracy. However, during motion, babblers often fly from one treetop to the next. When they arrive at a treetop, they stop for a few seconds and then continue to fly. We do not know if this stop is a rest or a scanning of the surroundings or both. In other cases, babblers climb up to a treetop for a few seconds and descend immediately. In order to avoid a situation in which every stay of several seconds on a treetop would be considered a sentinel bout (there are thousands of such cases), we classified as a sentinel bout a stay on a treetop of at least half a minute, and rounded out sentinel bouts to whole minutes.

When a sentinel detected a raptor or a terrestrial predator, it started to utter a series of calls, known in the literature as ‘alarm calls’ [37–43]. Such a series of calls last between a few seconds to two and a half minutes, and might be composed of one or two types of calls (described and analysed by [32,33]). The acoustic structure of the calls was not analysed in this work. The basic unit of alarm calls in this work is not an isolated call, but a series of calls. If the calls were stopped and renewed after a break of less than one minute, then they were considered to be part of the previous series. If the break lasted for more than 1 min, the first call after the break was considered to start a new series. During the study, we documented 609 series of calls, expressing, apparently, 609 encounters with predators. The raptor or the predator which caused the sentinel to call in 412 cases (67%) was identified. None of these cases was developed into pursuit or hunting, either because of the early detection of the predator or possibly also owing to our presence.

In order to know exactly where the babblers would be the following morning, it was necessary to locate them the previous day and follow them until they went to roost. A common factor for all the morning observations, therefore, is that the babblers experienced the presence of an observer the previous evening. A common factor for all the afternoon observations was the presence of an observer in the morning of the same day.

In order to neutralize the potential influence of the observer and standardize data collecting, we started to collect data when the babbler descended from its second sentinel bout. In other words, the first two sentinel events and the time periods before the first one and between the two were not included in the dataset analysed in this work. When data collection started, it continued exactly for 2 h.

The data presented here were collected over a period of 12 h of observation on each floater: 4 h per day (2 h in the morning and an additional 2 h in the afternoon), for three consecutive days. The sentinel behaviour of each floater was compared with its own sentinel behaviour as a group member in identical periods of time, during the year before leaving the group or being evicted, and out of breeding season. We collected data about the sentinel behaviour of 38 babblers over 228 days: 114 days as group members (38*3) and 114 additional days as floaters, and for 912 h: 456 h (38*12) as group members and 456 additional hours as floaters.

During the years of study, more than 38 babblers became floaters, but they were observed for less than 3 days, or we did not have information about their sentinel behaviour as group members. Therefore, data collected about them are not analysed in this study.

## 3. Statistical analysis

All analyses were performed using JMP (SAS Institute Inc., Cary, NC). The tests were performed on the sum values of 3 days' observations. All tests were two-tailed and were considered significant at *p* ≤ 0.05. Parametric tests were conducted as data fit the relevant assumptions of normality and homogeneity of variance. Linear-mixed models (LMMs) with REML methods were used to analyse behavioural data. For the dependent variables ‘sentinel activity duration’ and ‘number of sentinel events’, the fixed effects were membership (group members versus floaters), rank (dominants versus subordinates), sex (males versus females) and their two-way interactions. The model for ‘alarm calls' as the dependent variable included additionally the variable ‘accumulated sentinel duration’ as a covariant. Individual identity and residual were the random terms in all the models. The analysis yielded the significance of the fixed effects as well as the means and standard errors, and for random terms the variance components and their confidence interval.

## 4. Results

### 4.1. Sentinel activity of floaters compared with their sentinel activity when they were group members

All floaters acted as sentinels. Over 456 h of observation, 38 floaters performed 726 sentinel events, an average of 19.1 ± 3.5 events per individual per 12 h of observation. The duration of each event lasted on average 5.3 ± 3.1 min.

**Figure 1.**
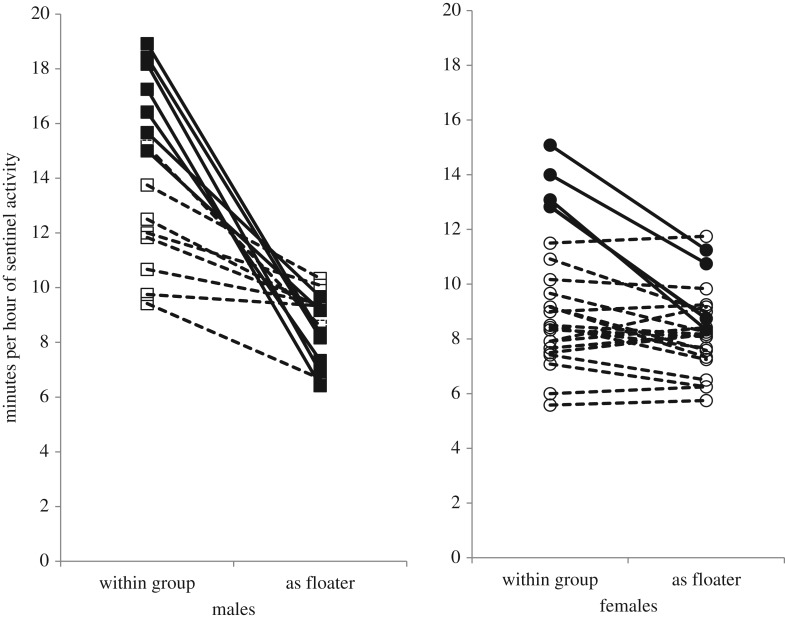
Sentinel activity duration of floaters versus their sentinel activity duration when they were group members: 15 males on the left panel (dark squares denote seven dominant males; open squares denote 8 subordinate males), 23 females on the right panel (dark circles denote four dominant females; open circles denote 19 subordinate females).

Figure 1 shows the sentinel activity of each individual as a floater compared with its sentinel activity when it was a group member.

Table 1 summarizes this data on sentinel activity duration per hour as well as on the number of sentinel events per hour.

**Table 1.** Minutes of sentinel activity per hour and number of sentinel events per hour.

	minutes of sentinel activity per hour		no. sentinel events per hour	
	as group members	as floaters	as group members	as floaters
all together (*n* = 38)	11.32 ± 4.89	8.44 ± 3.82	2.24 ± 0.80	1.59 ± 0.81
males (*n* = 15)	14.33 ± 4.79	8.60 ± 3.01	2.62 ± 0.80	1.63 ± 0.76
females (*n* = 23)	9.36 ± 3.86	8.34 ± 4.28	1.99 ± 0.69	1.56 ± 0.84
dominants (*n* = 11)	15.89 ± 4.55	8.64 ± 3.11	2.63 ± 0.87	1.57 ± 0.78
subordinates (*n* = 27)	9.46 ± 3.64	8.36 ± 4.08	2.08 ± 0.71	1.60 ± 0.82

Table 2 details the significance of the fixed effects on sentinel activity duration and on the number of sentinel events for the study.

**Table 2.** Significance of fixed effects for sentinel activity duration and for the number of sentinel events in the study (*n* = 38).

	sentinel activity duration LMM			no. sentinel events LMM		
	*F*	d.f.	*p*	*F*	d.f.	*p*
membership (within-group/floater)	234.72	1,35	<0.0001	98.27	1,35	<0.0001
rank (dominant/subordinate	34.40	1,34	<0.0001	3.67	1,34	0.064
sex (male/female)	10.38	1,34	0.003	15.54	1,34	0.0004
membership–rank interaction	73.90	1,35	<0.0001	6.54	1,35	0.015
membership–sex interaction	34.98	1,35	<0.0001	8.37	1,35	0.007
rank–sex interaction	2.37	1,34	0.133	0.36	1,34	0.552

Variance components: sentinel activity duration LMM: individual = 140.17 (44.5%), residual = 175.16 (55.5%); number of sentinel events LMM: individual = 0, residual = 13.9 (100%).

Together with the data in table 1, the analysis of membership–rank and membership–sex interactions in table 2 shows that upon becoming floaters, dominants reduced their sentinel activity significantly more than did subordinates, and males reduced their sentinel activity significantly more than did females.

Within the group, dominants' sentinel duration was significantly longer than subordinates' sentinel duration (LMM rank effect for membership = within-group: *F* = 90.47, d.f. = 1,57, *p* < 0.0001), and males' sentinel duration was significantly longer than females' sentinel duration (LMM sex effect for membership = within-group: *F* = 32.95, d.f. = 1,54, *p* < 0.0001). As floaters, in contrast, no significant difference was found between dominants and subordinates sentinel duration (LMM rank effect for membership = floater: *F* = 0.21, d.f. = 1,57, *p* = 0.645) or between males and females sentinel duration (LMM sex effect for membership = floater: *F* = 0.02, d.f. = 1,54, *p* = 0.880).

Similarly, within the group dominants guarded more frequently than subordinates (LMM rank effect for membership = within-group: *F* = 10.01, d.f. = 1,69, *p* = 0.002) and males guarded more frequently than females (LMM sex effect for membership = within-group: *F* = 23.58, d.f. = 1,69, *p* < 0.0001). As floaters, in contrast, no significant difference was found between the number of sentinel events of dominants and subordinates (LMM rank effect for membership = floater: *F* = 0.22, d.f. = 1,69, *p* = 0.643) or males and females (LMM sex effect for membership = floater: *F* = 0.82, d.f. = 1,69, *p* = 0.369).

### 4.2. Number of floaters' calls in reaction to approaching predators (‘alarm calls’) compared with their own calls when they were group-members

During 3851 min of sentinel activity (which were performed over 456 h of observation), 38 floaters reacted to approaching predators by uttering 261 series of ‘alarm calls’, an average of 6.9 ± 6.1 series per individual. Sentinel activity of the same individuals as group members (which were also performed during 456 h of observation) lasted 5164 min (34% more than the 3851 min of sentinel activity as floaters) and, during these 5164 min, they uttered 348 series of calls in reaction to approaching predators, an average of 9.2 ± 4.7 series per individual.

Table 3 presents the statistical analysis of the fixed effects on the number of alarm calls.

**Table 3.** Significance of fixed effects for the number of alarm calls in the study (*n* = 38).

	no. alarm calls LMM		
	*F*	d.f.	*p*
membership (within-group/floater)	2.60	1,58	0.113
rank (dominant/subordinate)	0.21	1,47	0.650
sex (male/female)	0.84	1,38	0.366
membership–rank interaction	0.04	1,51	0.851
membership–sex interaction	0.49	1,45	0.486
rank–sex interaction	0.16	1,35	0.694
accumulated sentinel duration	0.01	1,63	0.915

Variance components: individual = 13.33 (64.5%), residual = 7.34 (35.5%).

There was no significant interaction between accumulated sentinel duration and the number of ‘alarm calls’. Significant interaction was found neither between sex and rank nor between sex and membership, and not between rank and membership. No significant difference was found in the number of ‘alarm calls’ series between group members and floaters. No significant difference was found in the number of ‘alarm calls’ series between males and females and between dominants and subordinates (table 3).

## 5. Discussion

Studying the sentinel behaviour of floaters may help in better understanding the sentinel behaviour of group-living animals because, in some sense, this is the simplest social situation in which a group-living animal can live (although it is absolutely not simple to be a floater!). The environmental conditions created a natural experiment that made it possible to study this issue through observation only, with no active, harmful intervention.

### 5.1. Selfish versus other-regarding motives for sentinel activity

All floaters performed sentinel activity, and during sentinel bouts uttered ‘alarm calls’ at the same rate that they did when they were group members.

When a floater who lives alone acts as a sentinel, it does so not for the sake of its relatives or group-mates [2,11], not as part of parental care [12,44], not for group augmentation [45,46] and not even to improve its prestige in the eyes of its groupmates ([28], pp. 134–136; [13]). Floaters’ sentinel activity can derive only from a self-serving interest. This means that the direct benefits from sentinel activity, such as early detection of predators and/or signalling to the predators that they were revealed, outweigh the direct costs of sentinel activity, such as increased exposure to predators.

This suggests that selfish motives for sentinel activity exist also for group-living Arabian babblers, as proposed by Bednekoff [6] and supported by empirical evidence by Clutton-Brock *et al*. [20], Wright *et al*. [9,30] and Bednekoff & Woolfenden [47,48]. Our findings do not rule out the possibility that other-regarding motives are present as well in a group alongside the selfish motives which our study corroborates. In other words, the selfish interest for guarding may potentially be altered and complicated when sentinel activity is performed within a group. However, the need of floaters to guard for their own sake, and to utter vocalizations towards predators when they are completely on their own suggests that this selfish motive remains a fundamental incentive also when a babbler lives in a group.

### 5.2. Tracking conspecifics as one of the incentives for sentinel activity

During our study, only once was a floater discovered by the territory owners, attacked and managed to escape from its pursuers. In five other cases, the owners of the territory almost discovered the floater, but it hid itself and remained still and silent, deep in dense vegetation until the owners moved away.

This suggests that the location of the group owning the territory was a concern for floaters, and that detecting foreign conspecifics may be one of the incentives for floaters' sentinel bouts, in line with the claim of McGowan & Woolfenden [4] that Florida scrub jays use sentinel positions to detect territory intruders and repel them as well as the recent experimental results of Walker *et al*. [19] with white-browed sparrow weavers.

### 5.3. Potential reasons for the reduced sentinel activity of floaters

Floaters have a potential incentive to guard more than when they were group members in order to compensate for the absence of groupmates to share with them the burden of detecting predators. Our findings were, however, that floaters sentinelled less than group members. This suggests that floaters' motives to decrease sentinel activity outweighed their incentive to increase it.

One potential reason for floaters to decrease their sentinel activity could be the fear of being detected by the territory owners, as explained above. A second reason could be that foraging effectiveness is lower in a foreign territory, leaving less time for sentinel bouts. A third potential reason could be that floaters are weaker than when they were group members, leaving them with less energy for performing sentinel activity that requires forgoing foraging.

Each of the above potential reasons is also consistent with our finding that upon becoming floaters, dominants decreased their sentinel activity much more than did subordinates.

First, within a group, dominants have a higher incentive than subordinates to track conspecifics and to display ownership towards them. If same-sex conspecifics penetrate the group, then the dominant's breeding position might be challenged, whereas subordinates have less to lose from such a penetration. Guarding may be a means to detect and prevent such a penetration, and dominants have both more ability (following [6–9,30,47]) and more interest than subordinates in detecting and preventing such a penetration.

In addition, in the case of opposite-sex intruders, dominants would apparently benefit more than subordinates. Moreover, exposing oneself in a prominent position could be also used to display ownership towards foreign babblers who might enter the territory.

Second, within a group, dominants might be able to overtake good foraging spots detected by other group members, and scrounge food items found by others. As a result, the decrease in their foraging effectiveness when they become floaters is sharper than that experienced by subordinates. The foraging effectiveness of ex-dominant and ex-subordinate floaters may therefore be similar, hence leaving similar time for sentinel activity, as in our findings.

Third, it may be the case that dominants were weakened relatively more than subordinates upon becoming floaters, and hence the sharper decrease in sentinel activity of the former versus the latter.

### 5.4. The function of ‘alarm calls’

During sentinel activity, floaters uttered ‘alarm calls’ at the same rate that they did when they were group members. This suggests that ‘alarm calls’ are first and foremost part of predator–prey communication, aimed at signalling to predators that they are detected and hence unlikely to succeed in hunting the babbler ([28], pp. 3–9; [49]).

Within a group, this predator–prey communication by the sentinel may provide a *collateral* benefit as a warning signal to the sentinel's groupmates when they hear the sentinel's calls [4,8,11].

## 6. Conclusion

The sentinel behaviour is apparently aimed at gathering information about changes in the surroundings which may be important for the sentinel: approach of terrestrial predators, approach of aerial raptors, approach of a neighbouring group, presence of floaters, behaviour of group members, changes in food sources (such as blooming of plants), behaviour of other animals which may give hints about predators and food sources, changes in weather (approach of a whirlwind or a dust storm) as well as possibly other aspects, such as the behaviour of the person who is in their vicinity and observes them.

Some of these incentives for information gathering are relevant for everybody (e.g. the approach of a raptor), and some may motivate one individual more than others. Dominants are interested in displaying their ownership of the territory, their position in the group and their decisiveness to defend it (as suggested by [28] and [19]); subordinate adults may be interested in social changes in neighbouring groups; fledglings may be interested in the foraging success of group members in order to decide from whom it may be more beneficial to beg for food. In the presence of floaters in their territory, males have different interests than females, and dominants have different interests than subordinates. The different interests may lead to different sentinel regimes.

For an Arabian babbler group member, performing sentinel activity is apparently a consequence of a trade-off between hunger, defence from predators and the need to gather different kinds of information about the surroundings. The behaviour of other group members and the mutual relationships with them also influence the group member's sentinel behaviour. For a floater, this last component is apparently replaced by a trade-off between the dangers that the owners of the territory may pose and the need to gather information about them in order to assess the profitability of remaining in that territory: trying to join the group or escaping far away.

Despite the huge amount of research that has been invested in sentinel behaviour, we do not yet fully understand the motives that drive the babblers to stop foraging or to stop a social interaction and to climb up to the sentinel position. We do not know if sentinel behaviour is multitasked or one-target oriented. Except for detecting approaching predators, we do not know what the sentinel's main concerns are, what it sees and what it hears. Disentangling sentinel behaviour by its different motives and enriching our terminology accordingly about its multifaceted behaviour remains a challenge for future sentinel behaviour research.

## Data accessibility

Data are available at the Dryad Digital Repository: http://dx.doi.org/10.5061/dryad.4js7n [50].
